# Archaeology demonstrates sustainable ancestral Coast Salish salmon stewardship over thousands of years

**DOI:** 10.1371/journal.pone.0289797

**Published:** 2023-08-25

**Authors:** Meaghan Efford, Spencer Taft, Jesse Morin, Micheal George, Michelle George, Hannah Cavers, Jay Hilsden, Lindsey Paskulin, Doris Loewen, Jennifer Zhu, Villy Christensen, Camilla Speller

**Affiliations:** 1 Institute of Oceans and Fisheries, University of British Columbia, Vancouver, British Columbia, Canada; 2 Tsleil-Waututh Nation, North Vancouver, British Columbia, Canada; 3 Department of Archaeology, University of Oxford, Oxford, United Kingdom; 4 Department of Anthropology, University of British Columbia, Vancouver, British Columbia Canada; New York State Museum, UNITED STATES

## Abstract

Salmon are an essential component of the ecosystem in Tsleil-Waututh Nation’s traditional, ancestral, and contemporary unceded territory, centred on present-day Burrard Inlet, BC, Canada, where Tsleil-Waututh people have been harvesting salmon, along with a wide variety of other fishes, for millennia. Tsleil-Waututh Nation is a Coast Salish community that has called the Inlet home since time immemorial. This research assesses the continuity and sustainability of the salmon fishery at təmtəmíxʷtən, an ancestral Tsleil-Waututh settlement in the Inlet, over thousands of years before European contact (1792 CE). We apply Zooarchaeology by Mass Spectrometry (ZooMS) analysis to 245 archaeological salmon vertebrae to identify the species that were harvested by the ancestral Tsleil-Waututh community that lived at təmtəmíxʷtən. The results demonstrate that Tsleil-Waututh communities consistently and preferentially fished for chum salmon (*Oncorhynchus keta*) over the period of almost 3,000 years. The consistent abundance indicates a sustainable chum salmon fishery over that time, and a strong salmon-to-people relationship through perhaps 100 generations. This research supports Tsleil-Waututh Nation’s stewardship obligations under their ancestral legal principles to maintain conditions that uphold the Nation’s way of life.

## Introduction

**Fig 1:**
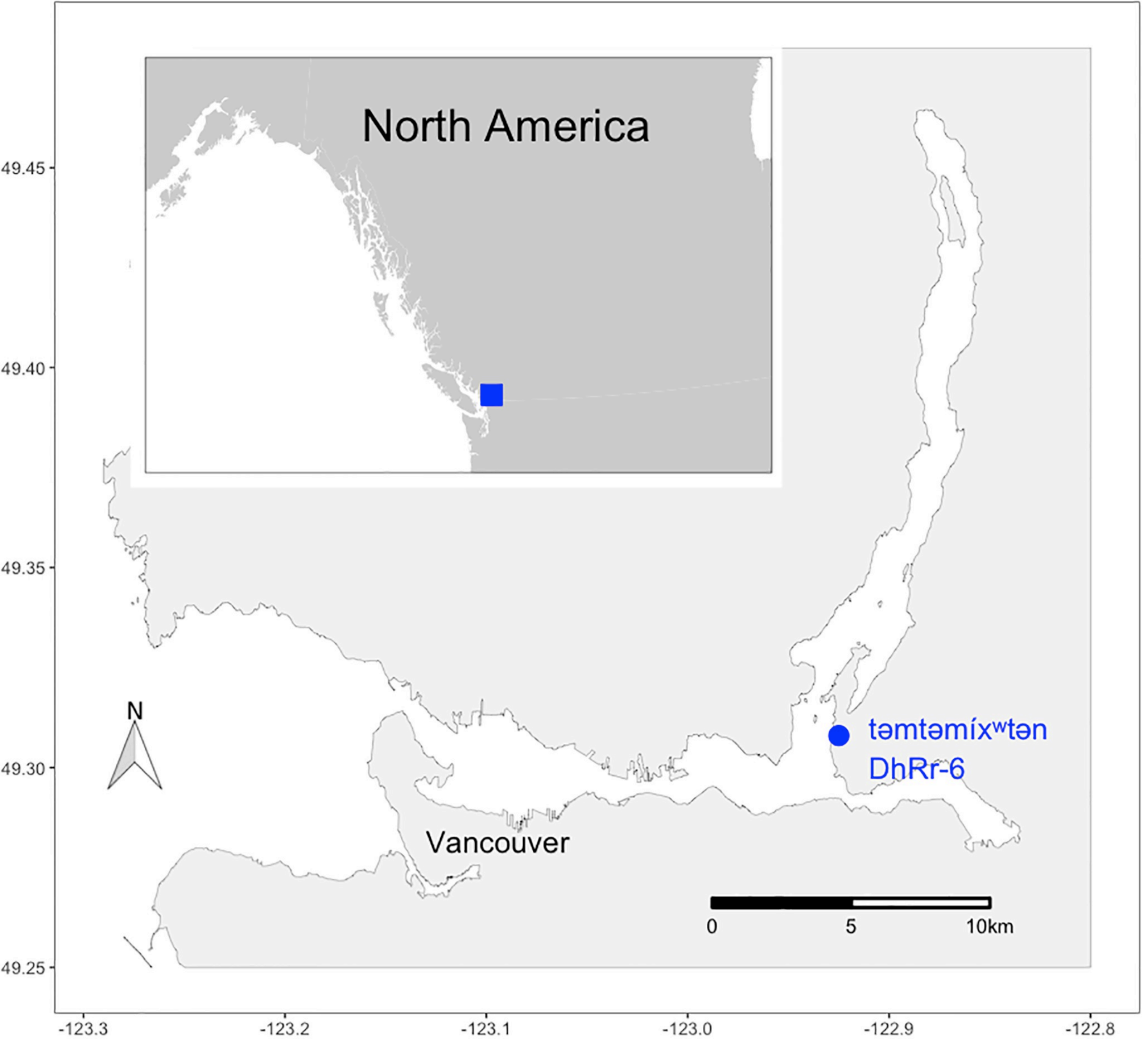
Map of Burrard Inlet with təmtəmíxʷtən identified. Created by V. Christensen 2023.

Tsleil-Waututh is a distinct, Indigenous Coast Salish nation whose ancestral and contemporary territory is centred on səlilwət, part of the inlet ecosystem that is now also known as Burrard Inlet, British Columbia, Canada [1] (Fig 1). Since European contact in 1792 CE, colonial development and resource extraction have driven dramatic change and development in Tsleil-Waututh territory to the extent that today it includes Canada’s busiest port and metropolitan Vancouver, a city of 2.5 million people. Consequent damage to and loss of vulnerable ecosystems, habitats, and animal and plant populations have impacted Tsleil-Waututh ways of life and greatly reduced their ability to harvest important traditional foods [1, 2]. Despite these impacts, the area is rich with archaeological evidence of Tsleil-Waututh’s relationship to the lands and waters throughout their territory and confirms Tsleil-Waututh oral histories and traditional knowledge of continuous connections to land, waters, plants, and animals [2–13].

*“Since time out of mind*, *Tsleil-Waututh have used and occupied Burrard Inlet and surrounding watersheds*. *Generations of Tsleil-Waututh People were brought up with the teaching*, *‘When the tide went out*, *the table was set*.*’ About 90% of our diet was once derived from Burrard Inlet and the Fraser River*, *but today the Inlet is unable to support our needs*.*”*

[14]

One place with exceptionally rich archaeological evidence in Tsleil-Waututh territory is təmtəmíxʷtən (Tum-tumay-whueton, meaning “lots of land”, or “biggest place for all the people”), a large and central ancestral settlement that was once home to several Tsleil-Waututh hereditary chiefs, making it an important settlement to Tsleil-Waututh communities [8, 9, 15]. The village is centrally located within the territory, situated in an ideal spot to take advantage of the diverse ecosystems, with salmon in the Inlet just as accessible as clam gardens, mountains, rivers, and forests [16]. The archaeological evidence here reflects the diverse local geography and indicates Tsleil-Waututh ancestors harvested across the ecosystems for millennia [17]. The village is at the corner of the Inlet as it moves north into Indian Arm (Fig 1), where Tsleil-Waututh’s primary traditional salmon fishery is located [1]. The ancestral Tsleil-Waututh diet consisted of salmon, herring, waterfowl, and clams, with berries, plant foods, fruits, deer and other land mammals, ling cod, anchovy, eulachon, and halibut, among others [1, 16]. Fishing and harvesting shellfish have been integral to ancestral Tsleil-Waututh communities [1, 8].

Indigenous communities from Alaska to California consider themselves salmon people, with salmon at the heart of their cultures, diets, traditions, and histories [18]. The rapid overexploitation and pollution of salmon populations by colonial settler communities that placed restrictions on Indigenous salmon stewardship and harvesting through violence has caused the loss of and damage to hundreds of Pacific salmon populations [18, 19]. Many Indigenous communities from this Pacific Northwest region are finding success in bringing back traditional salmon fishery practices and techniques that were banned by colonizing governments. Pairing traditional salmon fishery methods that have sustainability records spanning thousands of years with contemporary ecological science can provide unique, place-based, and sustainable methods to harvest from and protect contemporary salmon populations [19].

For many Coast Salish communities, salmon are and have always been people with communities and homes of their own, who would don their salmon skins to participate in their runs and voluntarily sacrifice their bodies to feed their human relations as part of their immortal lifespan, returning to the river to start anew [20, 21]. Salmon appeared numbering in the millions throughout coastal river systems in the Pacific Northwest and were key species in coastal ecosystems teaming with biodiversity across the food web [20]. The First Salmon Rites are an important part of cultures of the Coast Salish salmon peoples and have involved a celebration and welcoming of salmon to their annual runs and a return of the bones to the river [20]. Today, salmon remain an integral part of Coast Salish culture, including for the present day Tsleil-Waututh community, and this is evident in the archaeological evidence discussed in this research.

### The archaeology of təmtəmíxʷtən

Archaeological materials excavated from təmtəmíxʷtən include vast amounts of animal bones and shells (faunal material) deposited over millennia in a large formation called a shell midden [22] or cultural shell deposit, which provides a record of almost 3,000 years of history and occupation at this site [11]. One of the most abundant types of bones excavated from this midden are salmon vertebrae, demonstrating their crucial significance in the culture and sustenance of Tsleil-Waututh people and other Coast Salish communities [10, 18–21, 23–26].

Təmtəmíxʷtən is located on what is now also known as Belcarra Park at the junction of Burrard Inlet and Indian Arm (see Fig 1). Archaeologically, təmtəmíxʷtən (DhRr-6) is acknowledged as a large and very important settlement, central within Tsleil-Waututh territory and home to Tsleil-Waututh hereditary chiefs [7, 8]. Arthur Charlton led a large excavation project at təmtəmíxʷtən in 1971 as part of his Masters thesis research [4, 5]. Charlton’s work focused on culture-history based on the types of artefacts (stone, bone, and antler) and their representation of a time period not previously well understood archaeologically (400–1200 CE) in the region [5]. The focus of the collection and analysis efforts was primarily on stone and bone objects however large amounts of unprocessed midden samples and belongings, also known archaeologically as ‘artefacts’ [27–29], were also collected and stored at the Department of Archaeology at Simon Fraser University. Some effort was directed to quantifying mammal bones by Charlton [4, 5]. The analysis of the bone, antler, and lithic belongings resulted in Charlton’s presentation of two distinct, superimposed assemblages he called Belcarra Park I and Belcarra Park II.

The stratigraphy of the site is divided into three Stratigraphic Zones, with corresponding Cultural Units, Belcarra Park I and II [5]. Zone C includes Belcarra Park II, and Zone B includes Belcarra Park I, with Zone A including the sterile subsoil [5]. However, more recent work with many more radiocarbon dates provides a date range of up to 3500 years before present (uncalibrated yBP), falling within the Marpole Phase (500 BCE to CE 800) and the Gulf of Georgia Phase (CE 800 to 1792) [10, 11]. Charlton [5] recorded the size of the site as 150 m by 40 m, acknowledging that road construction had cut into the site and could have reduced the size before mapping could be done. Wave erosion is also cited by local residents as the cause of significant site erosion that further reduced the previous size of the site [5].

Stable isotope research on Ancestral Remains (i.e., human skeletal remains) from coastal sites from throughout British Columbia strongly indicate a marine-focused diet, with between 90–100% of food sources from marine and intertidal species [30–32]. That research included samples taken from Ancestral Remains found at təmtəmíxʷtən and dated to 340 BCE-350 CE (2290–1600 BP) [32], whose diet included about 96% marine protein [33]. Taken together, these research efforts indicate long-standing relationships to place and to the coastal ecosystem in particular, with the majority of the animals that sustained the human population coming from marine waters.

Teresa Trost contributed to the zooarchaeological efforts in Burrard Inlet with her Masters research, which focused on Say-umiton (Cove Cliff, DhRr-18) directly across the water (1.5 km) from təmtəmíxʷtən [34]. Say-umiton was likely a multi-seasonal village occupied for around 700 years before contact [34, 35]. Her analysis shows a wide variety of birds, mammals, fish, and invertebrate species, including an abundance of salmonids, Pacific herring (*Clupea pallasii*), spiny dogfish (*Squalus suckleyi*), perch (*Percidae spp*.), and Northern anchovy (*Engraulis mordax*) [34]. While her focus was not on təmtəmíxʷtən specifically, she does compare Say-umiton, təmtəmíxʷtən, Whey-ah-wichen (DhRr-8) and stl’álep (Tsawwassen, DgRs-2). The wide variety of fauna present at Cove Cliff speaks to the variety within the Tsleil-Waututh diet [34]

Nova Pierson added to zooarchaeological efforts at təmtəmíxʷtən and other sites in the Inlet with her research on resource use and marine conservation [17]. Her work focused on three sites: təmtəmíxʷtən (DhRr-6), Twin Islands (DiRr-16), and Say-mah-mit (Noons Creek, DhRq-1). At təmtəmíxʷtən, Pierson found 26 different fish groups in auger samples from these sites and calculated the ubiquity of all fishes. She also identified several invertebrate species and groups, several land and sea mammals, and numerous waterfowl [17]. Across all auger samples, the three most ubiquitous fish groups were Pacific salmon, Pacific herring, and Northern anchovy [17]. Salmon are as abundant or more abundant than herring or anchovy, and this pattern is consistent throughout the tested layers [17].

Pierson demonstrates that pre-contact Tsleil-Waututh fisheries were diverse, with salmon, herring, and anchovy as key groups, and a wide variety of other resident fish to supplement their diet [17]. This is in addition to other groups, like invertebrates, such as bivalves, mammals, and birds. Pierson argues that this diversification of the Tsleil-Waututh fisheries would have avoided depletion of any one species or group and would have contributed to the long-term sustainability of the fisheries [17]. Sustainable fisheries are key to ecosystem management and the health of the human and non-human communities, and management decisions would have to consider species across the local food web [17].

Assessing continuity of occupation is closely related to understanding relationships of people to place, and how those relationships traverse generations. Morin et al. [11] assessed continuity of the relationships between Tsleil-Waututh Nation and their territory in a meta-analysis of previously reported and newly obtained radiocarbon dates. They used 111 radiocarbon dates across 19 archaeological sites in eastern Burrard Inlet, including təmtəmíxʷtən. Their work demonstrates occupation within the Burrard Inlet area for over 3000 years before present, and evidence of continuity for at least 2250 years before present without apparent gaps or hiatuses more than about 30 years [11]. This remarkable continuity in local occupation strongly implies long-term ecological stability, and corresponding sustainable harvesting practices by the local inhabitants. This aligns with the identity of an Inlet-based Nation that Tsleil-Waututh Nation hold: indeed, their name “Tsleil-Waututh” means *People of the Inlet* [8, 11]. The earliest levels from təmtəmíxʷtən dated to about 1550 BCE, and the site was continuously inhabited up until post-contact times [11].

Some of the most recent archaeological efforts at təmtəmíxʷtən also focus on salmon remains [10, 12]. Ancient DNA (aDNA) analysis of salmon remains from a number of sites within the Inlet, including təmtəmíxʷtən, demonstrates that chum salmon (*Oncorhynchus keta*) were the most commonly harvested salmon species [10, 12], and that the inhabitants of təmtəmíxʷtən selectively harvested male chum (74.5% of salmon harvested) [10]. While much less common than chum, pink (*O*. *gorbuscha*), coho (*O*. *kisutch*), Chinook (*O*. *tshawytscha*, and sockeye (*O*. *nerka*) were also recovered. A 1986 catalogue of the salmon streams of the area report chum, pink, and coho salmon as the only species of any abundance in the streams of Burrard Inlet in 1947–1985 [36].

A recent historical ecology analysis of Burrard Inlet zooarchaeological assemblages shows this variety at not only təmtəmíxʷtən, but other archaeological sites in the Inlet [37]. Salmon, herring, eulachon, anchovy, smelt, and other fish species are present in abundance [37]. After European contact and throughout the colonization of Vancouver, dynamite fishing, overfishing, ecological damage, and pollution have caused devastating collapses in herring, smelt, and eulachon populations in the order of 99% [37]. Colonial salmon canneries imposed industrial fishing pressure on the salmon populations in the Inlet and elsewhere, reducing populations that once provided a cultural and dietary pillar for Tsleil-Waututh communities [12, 37].

### Salmon in Burrard Inlet

Several salmon species have been important both ecologically and culturally throughout the Salish Sea, including the inlet systems of the lower mainland of what is now known as British Columbia, Canada. Sockeye, coho, pink, chum, and Chinook salmon [38–40] can be found throughout this region. Since these species of salmon vary significantly in their appearance, behaviour, spawning season, and nutritional value to humans [41], determining which of these species have been the preferred species to fish can provide insight into the relationships between Tsleil-Waututh Nation’s ancestral communities and the salmon populations that moved through the Burrard Inlet watershed [39].

The sustainability of harvesting a specific group over time can be measured by assessing how consistent and abundant the species are in the archaeological record [26, 42–45]. If there are no valleys following a peak in abundance, no decline in abundance over time towards the present, and no sudden changes in targeted species, we assume that the harvest would have been sustainable. Taphonomic processes, such as acidic ground water eroding bone, shell, and stone, compression of the ground exacting pressure, and burrowing animals disrupting the ground, all impact zooarchaeological remains differentially depending on their fragility, density, and shape and “…selectively deleted portions of the original record” [46]. The longer material spends in the ground, the more of an impact taphonomic processes will have, and the greater attrition these remains will experience. However, present day activity, such as construction and human movement, will impact the materials closer to the surface. Burrowing animals and environmental changes can impact the material throughout the cultural shell deposit. Based on this expectation, in order to assess the Tsleil-Waututh salmon fishery as sustainable over time, the salmon remains would have to follow this expected stasis in abundance over the time period analysed, with no peaks, valleys, gradual declines, or sudden changes in target species moving towards the present. Sarah Campbell and Virginia Butler demonstrate that archaeology can offer a unique and comprehensive view into the sustainability and intensity of salmon fisheries in the past [47, 48]. Their work shows that there must be “resilience in the ecological-human system” for stability in salmon fisheries to be maintained over hundreds or thousands of years [48]. Their work does not dive into species-level analysis of fishery resilience over time, however. As a result, it can be challenging to detect or demonstrate sustainable practices relating to particular salmon species or populations.

Along with marine and terrestrial mammals, birds, invertebrates, and other fishes, salmon have been some of the most important populations of interest to Tsleil-Waututh communities for millennia, for food, social, and ceremonial purposes [1, 8]. The Tsleil-Waututh subsistence economy relied upon salmon, herring, clams, and marine birds as pillars of the diet [16]. The Inlet is an important habitat for chum salmon, and archaeological and ecological evidence demonstrates that chum could be found in abundance in the Inlet until recently [10]. The nearby Fraser River provides important habitat to sockeye [24], pink [24], chum [24], Chinook [24], and coho [24] salmon. Both oral testimony [1] and archaeological evidence [10] point to chum salmon as being particularly important for Tsleil-Waututh communities over the millennia.

### Zooarchaeology by mass spectrometry

Collagen peptide mass fingerprinting (Zooarchaeology by mass spectrometry—‘ZooMS’) is a rapid, cost-effective, minimally destructive, and potentially non-invasive method for taxonomic identification [49–51]. In the ZooMS method, collagen is extracted from archaeological bone and subjected to enzymatic digestion, which cleaves proteins at specific amino acid sites producing a characteristic mixture of peptides. The peptides are analysed through Matrix Assisted Laser Desorption/Ionisation Time of Flight Mass Spectrometry (MALDI-ToF-MS). Differences in the collagen amino acid sequence of various species produce distinct ‘peptide mass fingerprints’ based on their respective mass-to-charge (m/z) ratios, and mass spectra from unknown samples can be taxonomically identified through comparison with collagen fingerprints from a known reference database. ZooMS has been utilized to taxonomically identify salmon remains in previous studies [39, 49, 50].

### Objectives

In a recent study by Morin et al. [10], aDNA analysis of salmon remains from five archaeological sites in Burrard Inlet revealed that chum salmon were by far the most commonly harvested salmon species by Tsleil-Waututh Nation communities during a pre-contact period of 390 BCE to CE 1600. Based on that research, this project applied Zooarchaeology by Mass Spectrometry (ZooMS) to further assess the taxonomy of salmon at təmtəmíxʷtən, one of the five sites in the previous study [39, 49]. Based on the analyses by Morin et al. [10], we expected that chum salmon species would be more abundant and ubiquitous than other species. Additionally, we use eight new radiocarbon dates derived from Charlton’s earlier excavations to track change through time in salmonids. The species identification and radiocarbon results will provide an opportunity to determine if there is a temporal pattern that can be discerned in the relative abundance of salmon species in the təmtəmíxʷtən shell deposit.

Due to this importance of salmon to past and present Tsleil-Waututh people, culture, and diets [10], this research seeks to assess the continuity and sustainability of the salmon fishery at təmtəmíxʷtən before changes brought by colonization began. We analyse species-level fishery resilience through deep time. This archaeological investigation into salmon vertebrae from təmtəmíxʷtən can help reconstruct stewardship practices and human-to-environment relationships of Tsleil-Waututh community and their territory through deep time. This work is in service to and part of Tsleil-Waututh Nation’s Cumulative Effects Monitoring Initiative, which serves the Nation’s stewardship responsibilities and rights [16].

## Materials and methods

**Fig 2:**
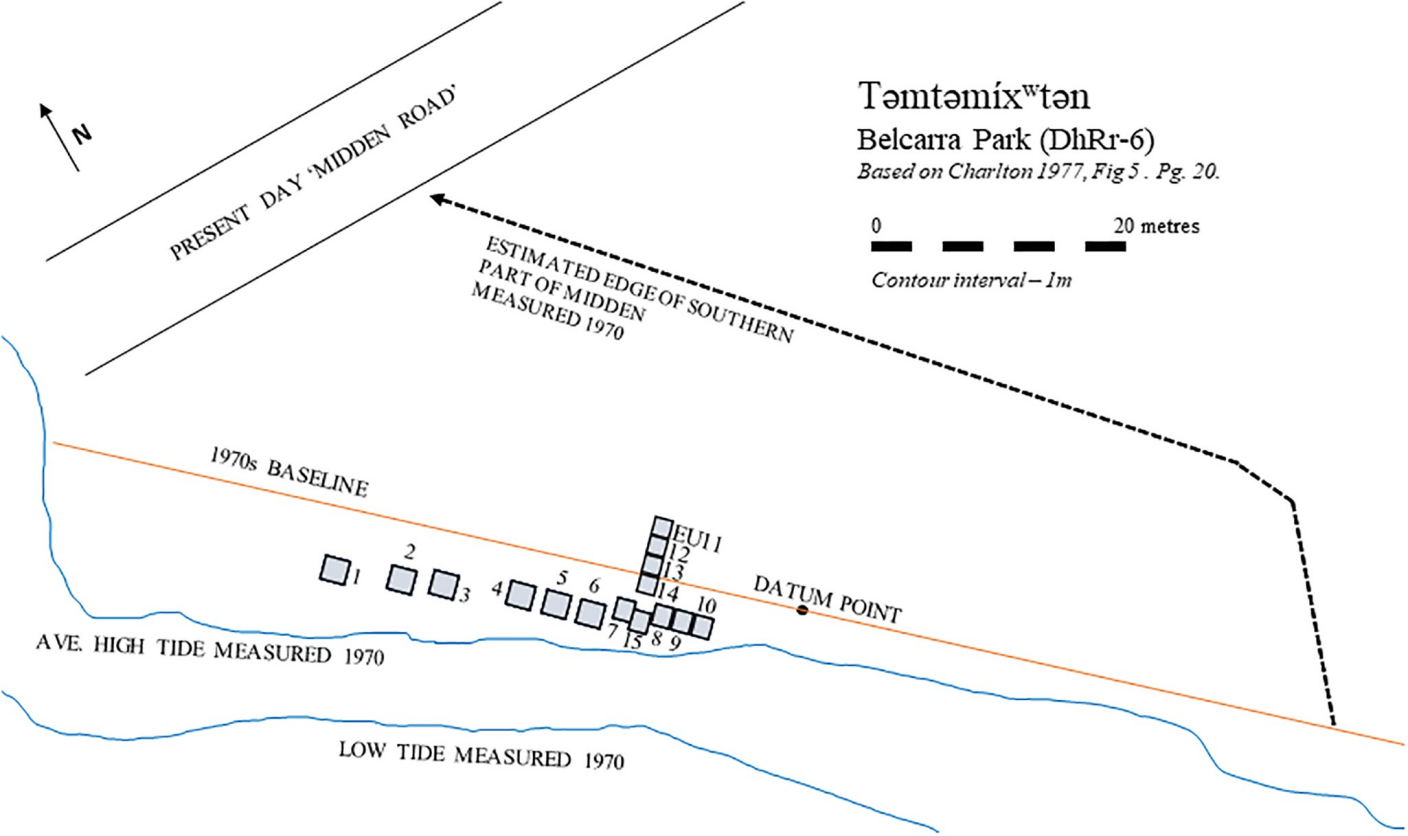
Site map of təmtəmíxʷtən (DhRr-6), based on original site map: Charlton 1977, Fig 5, Pg. 20. Present day Midden Road leading to Belcarra Pier is noted.

We analysed archaeofauna collected by Charlton [4, 5] during his previous (1971) excavations at təmtəmíxʷtən. Salmon vertebrae were taken from two excavation units, Unit 116–118 N, 0–2 W (see Table 1) and Unit 116–118 N, 4–6 W (see Table 2). The two Excavation Units (EUs) discussed here are labelled as Units 116–118 N, 0–2 W and 116–118 N, 4–6 W. Based on the original site map, adapted here in Fig 2 for clarity, these two EUS are #14 and #15. To determine this, we consider the original labels as measurement instructions, to be measured from the Base Line referenced in the map Fig 2. The original publications for the initial archaeological work at this site do not match EU 1–15 with the labels on the boxed archaeological material [4, 5]. As it is not clear, we reference Units 116–118 N, 0–2 W and 116–118 N, 4–6 W with those labels rather than Unit numbers. Permission for the destructive analysis of the archaeological samples is covered under a research Agreement between Tsleil-Waututh Nation and the University of British Columbia, signed on March 16, 2020 (UBC Ref. M20-00122). No permits were required for the described study, which complied with all relevant regulations. Salmon samples investigated in this study are accessioned in Simon Fraser University’s Museum of Archaeology and Ethnology, Burnaby, BC, Canada; all specimen and provenience information is provided in S1 Table in S1 File.

**Table 1:** Sampling for excavation unit 116–118 N, 0–2 W.

Level (cm)	Context	ZooMS samples: salmon	NISP fish vertebrae	Radiocarbon samples
0–10	Level 1		2	S02-1
10–20	Level 2	L2 B1-6 (N = 6)	9	
20–30	Level 3	L3 B1-14 (N = 14)	14	
30–40	Level 4		28	
40–50	Level 5	L5 B1-25 (N = 25)	230	
50–60	Level 6		1000	S02-6
60–70	Level 7		1600	
70–80	Level 8	L8 B1-25 (N = 25)	3000	
80–90	Level 9		220	
90–100	Level 10		54	
100–110	Level 11	L11 B1-25 (N = 25)	51	S02-11
110–120	Level 12		22	
120–130	Level 13	L13 B1-20 (N = 20)	220	
130–140	Level 14		7	
140–150	Level 15		114	
150–160	Level 16	L16 B1-20 (N = 20)	45	
160–170	Level 17		2	S02-17
TOTALS		135	6713	4/8

**Table 2:** Sampling for excavation unit 116–118 N, 4–6 W.

Level (cm)	Context	ZooMS samples: salmon	NISP fish vertebrae	Radiocarbon sample ID
0–10	Level 1		29	
10–20	Level 2		33	
20–30	Level 3	L3 V1-20 (N = 20)	35	S46-3
30–40	Level 4		85	
40–50	Level 5	L5 V1-20 (N = 20)	280	
50–60	Level 6		93	
60–70	Level 7		27	S46-7
70–80	Level 8	L8 V1-20 (N = 20)	218	
80–90	Level 9		197	
100–110	Level 11		350	
110–120	Level 12	L12 V1-20 (N = 20)	401	
120–130	Level 13		323	
130–140	Level 14		421	S46-14
140–150	Level 15	L15 V1-20 (N = 20)	200	
150–160	Level 16		46	
160–170	Level 17	L17 V1-10 (N = 10)	135	
170–180	Level 18		8	S46-18
TOTALS		110	2881	4/8

### Sampling and ZooMS analysis of salmon remains

An initial morphological identification provided a general overview of the range of fish, mammal, and bird species, with thousands of salmon vertebrae dominating the bone assemblage. See Tables 1 and 2 for the NISP of fish vertebrae from each excavated level. Approximately 80% of the fish vertebrae from this assemblage are salmon. Salmon vertebrae were selected for collagen fingerprinting to determine the relative abundance of salmon species. Individual vertebrae were chosen due to their completeness and the confident identification as salmonids, with an attempt to include a range of vertebral size. The majority of the vertebrae in all levels are salmon, with herring and spiny dogfish (*Squalus suckleyi*) appearing in most levels and other fishes appearing sporadically. This could be due to collection bias or differential preservation, as well as an overwhelming preference for specific species or groups. EU 116–118 N, 0–2 W has a large concentration of 5600 salmon vertebrae around 50–80 cm below surface, all of which were gathered on their own in large paper bags, indicating that this concentration was deliberately sampled and is due to field collection methods (see Tables 1 and 2).

All samples were analysed within the Ancient DNA and Proteins (ADαPT) Facility in the Department of Anthropology at the University of British Columbia. A total of 245 fish vertebrae were analysed using the method published in Buckley et al. (2009), modified as described in Richter et al. [39] (S1 Table in S1 File). Briefly, ca. 10–30 mg of bone was subsampled and demineralized 0.6 M HCl at 4°C. Samples were rinsed in 200 μL of 0.1 M NaOH to remove humic compound, then rinsed three times in the same volume of 50 mM ammonium bicarbonate solution (NH4HCO3) pH 8.0 (AmBic). Samples were gelatinized through incubation in 100 μL of AmBic at 65 °C for 1 h, before being enzymatically digested overnight at 37 °C in 0.4 μg of trypsin. Digested samples were acidified to 0.1% trifluoroacetic acid (TFA) and purified using Pierce^™^ 100 μL C18 tips (ThermoFisher). One microliter of α-cyano-hydroxycinnamic acid (matrix) was added to 1 μL of collagen extract and spotted in triplicate with onto a 384 spot MALDI target plate alongside calibration standards. MALDI-TOF was conducted on a Bruker Ultraflex III mass spectrometer with a Nd:YAG smart beam laser, with a SNAP averaging algorithm was used to obtain monoisotopic masses (C 4.9384, N 1.3577, O 1.4773, S 0.0417, H 7.7583). Triplicate spectra were averaged, and visually inspected using mMass software [52] to identify diagnostic markers published in Richter et al. [39]. Raw spectra have been deposited into the Dryad data repository (https://doi.org/10.5061/dryad.hqbzkh1mt). Spectra were initially compared to the list of shared salmonid markers to confirm the vertebrae were derived from Pacific salmonids (S2 Table in S1 File), and subsequently to diagnostic markers for species identification (S3 Table in S1 File). Samples which had diagnostic markers which appeared but below a signal-to-noise ratio of 6 were listed as ‘probable’ identifications.

### Radiocarbon dating

We sampled eight ungulate long bone fragments recovered from Charlton’s excavations for radiocarbon dating. We sampled one from the top, two from the middle, and one from the bottom of each the excavation unit from Charlton (1980) that we reanalyzed (see Tables 1 and 2) with the goal of obtaining dates from earliest, middle, and latest contexts. Samples were radiocarbon dated at the A. E. Lalonde AMS Laboratory at the University of Ottawa and calibrated using OxCal v4.2.4 [53] and the IntCal20 calibration curve [54].

## Results

### Radiocarbon dating results

**Fig 3:**
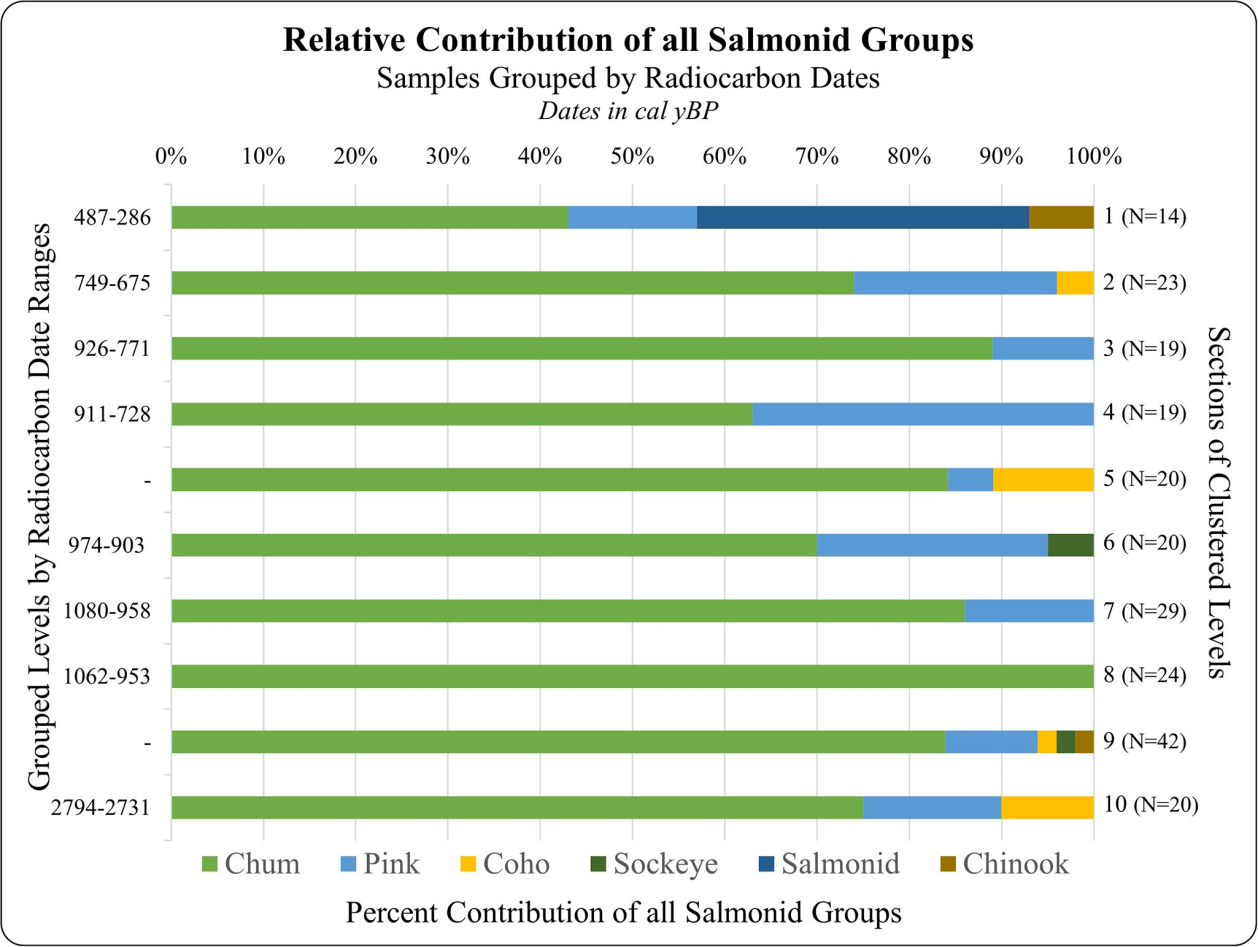
Grouped salmon samples by corresponding level and radiocarbon date ranges. All samples from təmtəmíxʷtən (DhRr-6), from Excavation Units 116–118 N, 0–2 W and 116–118 N, 4–6 W. Levels are grouped by associated radiocarbon dates. Missing dates on left Y axis indicates no radiocarbon date for that cluster of levels. Most recent samples are on the top. Dates are in calibrated years before present.

Eight radiocarbon assays were obtained, listed in Tables 3 and 4 as calibrated years before present (Cal yBP) (See S4 Table in S1 File for uncalibrated dates). The dates fall within expected ranges, based on previous radiocarbon dating efforts performed at təmtəmíxʷtən [7]. Since there are four dates per Excavation Unit, samples from the top, middle, and bottom of the Units (see Tables 3 and 4), the levels from both Units can be aligned to allow for a more in-depth analysis from this site, as seen in Fig 3.

**Table 3:** Radiocarbon dating sampling and results for excavation unit 116–118 N, 0–2 W at təmtəmíxʷtən (DhRr-6).

Level (cm)	Context	Radiocarbon Sample ID (Lab ID)	Radiocarbon dates (Cal yBP, 95.4% probability)
0–10	Level 1	S02-1 (UOC-16082)	487–286 (93.9%) 166–156 (1.6%)
10–20	Level 2		
20–30	Level 3		
30–40	Level 4		
40–50	Level 5		
50–60	Level 6	S02-6 (UOC-16083)	774–757 (5.9%) 749–675 (89.6%)
60–70	Level 7		
70–80	Level 8		
80–90	Level 9		
90–100	Level 10		
100–110	Level 11	S02-11 (UOC-16084)	1062–953 (90.1%) 947–932 (5.4%)
110–120	Level 12		
120–130	Level 13		
130–140	Level 14		
140–150	Level 15		
150–160	Level 16		
160–170	Level 17	S02-17 (UOC-16085)	2848–2810 (15.5%) 2794–2731 (79.9%)
TOTALS		4/8	

**Table 4:** Radiocarbon dating sampling and results for excavation unit 116–118 N, 4–6 W at təmtəmíxʷtən (DhRr-6).

Level (cm)	Context	Radiocarbon sample ID (Lab ID)	Radiocarbon dates (Cal yBP, 95.4% probability)
0–10	Level 1		
10–20	Level 2		
20–30	Level 3	S46-3 (UOC-16086)	926–771 (92.9%) 760–746 (2.5%)
30–40	Level 4		
40–50	Level 5		
50–60	Level 6		
60–70	Level 7	S46-7 (UOC-16087)	911–728 (95.4%)
70–80	Level 8		
80–90	Level 9		
100–110	Level 11		
110–120	Level 12		
120–130	Level 13		
130–140	Level 14	S46-14 (UOC-16088)	1046–1039 (0.7%) 974–903 (86.0%) 868–824 (7.6%) 811–801 (1.2%)
140–150	Level 15		
150–160	Level 16		
160–170	Level 17		
170–180	Level 18	S46-18 (UOC-16089)	1177–1163 (4.7%) 1123–1090 (8.3%) 1080–958 (82.5%)
TOTALS		4/8	

### ZooMS results

230 of the 245 samples that were analyzed were identified as Pacific salmon. Four were identified as non-salmon fish and 18 could not be confidently identified. Of the 230 salmon, 180 were identified as chum, 33 as pink, six as coho, two as chinook, and two as sockeye salmon; of these 113 of the chum were given ‘probable’ identifications, as were three pink and one coho (Fig 3). Five vertebrae could not be identified to species but were confirmed to be salmon based on shared markers across Pacific salmon. The samples that were only identifiable as either salmonid or No ID (see Table 5) displayed poor collagen preservation, and insufficient shared or diagnostic salmonid markers for confident taxonomic identification. These results mirror the relative proportions from the previous aDNA results [10], which suggests that ZooMS is a feasible method for the taxonomic identification of Pacific salmon and can be a reliable, cost-effective option for future research efforts. This is the largest taxonomic identification effort for salmon remains in the Pacific Northwest using the ZooMS approach or aDNA.

**Table 5:** Compared results from Morin et al 2021 and 2022 ZooMS results performed for this report. * Indicates salmon total from təmtəmíxʷtən only, excluding other sites in 2021 study.

	2022 ZooMS		aDNA [10]	
	N	% of Salmon Total	N	% of Total
Total	245		123	
Salmon total	231		*55	
Chum salmon	181	78.35%	41	74.50%
Pink salmon	32	13.85%	12	21.80%
Coho salmon	6	2.60%	0	0.00%
Sockeye salmon	2	0.87%	1	1.80%
Chinook salmon	2	0.87%	1	1.80%
Salmon	8	3.46%	-	N/A
No ID	14	6.06%	-	N/A

The abundance of chum salmon remains consistent across time (Fig 3). This is clear across ten sections of clustered column sample levels from two Excavation Units, which are aligned using the eight new radiocarbon dates. Two sections of clustered column samples are not associated with a radiocarbon date but are instead relatively dated using the clustered groups above and below them. Chum salmon are by far the most abundant and remain so over time. The average contribution of all salmon groups across all levels are as follows: 78% chum salmon, 14% pink salmon, 3% coho salmon, 1% sockeye salmon, and 1% Chinook salmon, with 3.5% of the samples identified as salmonids. A chi-square test of independence revealed no significant difference between species frequencies throughout the tested levels, X^2^ (36, N = 225) = 49.1, p = 0.07.

## Discussion

This research focuses on using zooarchaeological remains from təmtəmíxʷtən to investigate and contextualize Tsleil-Waututh stewardship of chum salmon. We focus on the relationship between the Tsleil-Waututh people living at təmtəmíxʷtən and the chum salmon population that spawns the rivers and streams draining into Burrard Inlet over time. Tsleil-Waututh salmon relations are an important part of the Inlet ecosystem, and the chum salmon population specifically has provided nutrition to Tsleil-Waututh people for millennia. While the 2021 aDNA results [10] are very similar (see Table 5), suggesting that the relative proportion of salmon species in the archaeological record is accurately represented, this study offers a higher resolution analysis over a longer period of time. These results suggest that chum salmon and pink salmon were by far the most abundant salmon species in the Inlet pre-contact compared to Chinook salmon and sockeye salmon. In the present day, chum and pink salmon, along with coho salmon, are the only salmon species that are present in Burrard Inlet in any abundance [36], and this suggests that the Chinook and sockeye salmon remains we see in the midden could be from the nearby Fraser River. Level Group 9 in Fig 3 includes a longer range in time, and so more samples, than any of the other Level Group (43 versus 29 or fewer). These results, although obtained from only two excavation units, are supplemented by the larger zooarchaeological record at təmtəmíxʷtən [10, 12, 17] and nearby sites [17] which attests to a consistent and sustainable exploitation of salmon over millennia. Pierson’s zooarchaeological analysis of auger samples from təmtəmíxʷtən demonstrate the ubiquity of salmon (in addition to herring and anchovy) within all the tested levels, with salmon bones representing 30–43% of the total NISP in the three auger samples [17]. The ubiquity and abundance of Pacific salmon though time, in addition to Pacific herring and anchovy, are interpreted as evidence of a highly specialized and sustainable fishery. Our molecular results confirm that for Pacific salmon, this fishery focused specifically on chum salmon.

Chum salmon are particularly susceptible to overfishing compared to other salmon species, and the maximum sustainable yield (MSY) of chum salmon appears to be 30% compared to the 40–70% range for other species (Carl Walters, personal communication 2023). The persistence of the chum fishery over generations speaks to a sustainable harvest of a sensitive population, which would likely require intentional sustainable harvest practice. Our results confirm the long-term successfully sustainable harvest of a target species over many generations.

### Ecosystem management

The colonial and early archaeological narratives surrounding the Indigenous communities of the Pacific Northwest emphasized the idea that Indigenous peoples in the Salish Sea were complex hunter-gatherers, not cultivators or ecosystem managers [29, 55–58]. These narratives reinforced the idea that the land- and waterscapes in the region, and the plants and animals that made the region their home, were untouched and unclaimed, ready to be adopted and cultivated by European settler communities. Despite these arguments persisting through early colonization and into the present day, archaeological efforts to understand past environments and human-to-environment relationships in the region have demonstrated what Indigenous communities have been asserting: these land- and waterscapes are in fact highly managed spaces, with ancestral community ties and responsibilities to steward and manage [29, 55–59]. Examples of these managed spaces include clam gardens [58, 60], forest gardens [56, 57], and fisheries [10–12, 29], including salmon fisheries [61]. This work contributes to our understanding of pre-contact salmon fishery management and provides a strong example of a sustainable Tsleil-Waututh chum salmon fishery.

### Salmon stewardship

Along with marine and terrestrial mammals, birds, invertebrates, and other fish, salmon have been one of the most important populations of interest to Tsleil-Waututh communities for millennia, and were used for food, ceremony, and community purposes [1, 8]. The Indian River at the northernmost end of Burrard Inlet is an important spawning habitat for chum salmon, as are the Seymour and Capilano Rivers, and archaeological and ecological evidence demonstrates that chum could be found in abundance in the Inlet [10, 36]. The nearby Fraser River provides important habitat to sockeye [24], pink [24], chum [24], Chinook [24], and coho [24] salmon. Both oral histories [1] and the archaeological evidence presented here and in Morin et al., (2021a) identify chum as being particularly important for Tsleil-Waututh communities over the millennia. The consistency of the comparative abundance of salmon species across time suggests that Tsleil-Waututh Nation’s chum salmon fishery was a stable and sustainable salmon fishery, maintained over the course of almost 3,000 years (Table 3). We would expect to see a drop off in the abundance of chum salmon over time, or a switch to another species as the most abundantly harvested, if the fishery had been unsustainable, or if there had been a major ecological impact. Instead, through this work as others, we see consistent use of salmon, particularly chum salmon, as well as herring, anchovy, and eulachon over millennia [10–12, 17, 34]. For example, Virginia Butler’s 2200-year analysis of faunal resource use in eight sites from the Columbia River region noted a decline in the use of large salmon and fish species around AD 200, which may be associated with human-induced population declines, before fisheries stabilized. The continued abundance of salmon within the larger zooarchaeological assemblage at təmtəmíxʷtən emphasizes the consistent importance of salmon through time, while our high-resolution species profile obtained through ZooMS confirms the long-term dependence on chum specifically. While this study confirms that Indigenous management practices were successful in maintaining a resilient chum fishery, additional analyses assessing changes in the genetic diversity, spawning age or size of chum through time may reveal more fine-grained impacts harvesting pressures [62–64].

This collection of salmon vertebrae samples represents the largest archaeological salmon assemblages identified to species in the Pacific Northwest. Previous DNA-based sex identification of salmon in the same study region [12], indicates that the chum salmon harvest at təmtəmíxʷtən was sex-selective, and therefore more likely to happen during spawning in the late fall as they swim upstream, when the male salmon are easier to identify in the water due to their dramatically changed appearance, allowing the females to continue upstream [12]. The choice to focus harvesting on male chum salmon, and the evidence of sustained abundance of chum salmon over thousands of years, is indicative of a sex-selective resource management strategy [10, 12]. The previous DNA evidence was limited to samples dating to approximately 2300–1000 BP [12], while this work expands that temporal range at təmtəmíxʷtən specifically to approximately 2800–300 BP, and greatly increases the sample size. Our expanded sample demonstrates that chum was the dominant salmon harvested at təmtəmíxʷtən for 2500 years, during all sampled periods and locations.

The ability to identify salmon remains to species provides new data with which archaeological efforts can understand past environments and the people who make those environments home. Previous efforts in Burrard Inlet utilized aDNA [10, 12, 34, 65]. This approach using the less expensive ZooMS approach allows for an expanded sample size. This study utilises the largest sample of salmon vertebrae in the Pacific Northwest region that we know of, which has allowed for a robust data set when paired with radiocarbon dates and previous work. This study offers species-level analysis of salmon resilience and sustainability through time. Understanding the species-specific relationships between people, animals, and place can be instrumental in reconstructing past environments and understanding human subsistence and food security.

## Conclusions

Salmon were and are important to Coast Salish communities and ecosystems, and indeed have been a staple of Coast Salish diets and culture for millennia [10, 18–21, 23–25, 47, 48]. Salmon as central to diets can be seen throughout the Pacific Northwest through zooarchaeology [48]. The pre-contact Tsleil-Waututh community at təmtəmíxʷtən had a long-standing relationship with the chum salmon population in Burrard Inlet. This significance is reflected in the archaeological record over 2500 years and remains consistently abundant over that time. The archaeological evidence collected over the past decades reinforce arguments that salmon, and particularly chum salmon, have been important to Tsleil-Waututh people, culture, sustenance, and the local ecology for millennia. Further, the evidence shows a sustainable chum fishery at təmtəmíxʷtən spanning 2800–300 BP. This work is the largest ZooMS analysis of salmon on the Pacific Northwest Coast and contributes to community-driven archaeology based in Tsleil-Waututh Nation. Archaeological evidence like that which we discuss in this study provides data that can be used to inform resource and ecosystem management decisions.

Pacific salmon populations are vulnerable to anthropogenic driven changes to their environments, including warming oceans due to climate change, ocean acidification, and pollution [66, 67]. It is evident that settler colonial fishery practices have had devastating impacts on Burrard Inlet fisheries [37]. Burrard Inlet has experienced rapid and extensive changes since the start of colonization in the mid 1800s [2, 13]. This study shows that the Tsleil-Waututh chum salmon fishery was sustainable for thousands of years, and there are specific resource management choices that have contributed to that sustainability, including sex-selective harvesting [12]. Tsleil-Waututh Nation is well positioned to continue salmon resource management in their territory and work towards a sustainable salmon fishery once again. Focusing on chum habitat enhancement and protection would allow the Nation to reduce negative impacts on the chum salmon population and increase food security using a traditionally important food source.

## Supporting information

S1 File (XLSX)
